# The gender wage gap in the French veterinary labor market

**DOI:** 10.3389/fvets.2022.1001012

**Published:** 2022-11-14

**Authors:** Mehdi Berrada, Youba Ndiaye, Didier Raboisson, Guillaume Lhermie

**Affiliations:** ^1^CIRAD, UMR ASTRE, Montpellier, France, ASTRE, CIRAD, INRAE, Univ Montpellier, Montpellier, Université de Toulouse, ENVT, Toulouse, France; ^2^Department of Production Animal Health, Faculty of Veterinary Medicine, University of Calgary, Calgary, AB, Canada

**Keywords:** econometrics, economics, gender wage gap, public policy, veterinary labor market, France

## Abstract

Among the most important recent changes in the veterinary profession is the increasing percentage of women. Understanding trends driving the veterinary labor market is important to enable leaders in the veterinary profession and policy makers to plot strategic actions that will improve the profession. The objective of this paper was to estimate the gender pay gap in the veterinary labor market. We analyzed data from an anonymous online survey conducted in France in 2021 by the veterinary practitioner union. We assessed the gender gap pay using two regression approaches, ordinary least squares method and Blinder-Oaxaca decomposition, while controlling for all other variables (*ceteris paribus*). We provided evidence that male veterinarians earned wages approximately 9.3% higher (controlling for all other variables). This difference represented the “unexplained variance” that may be due to gender discrimination or unobserved characteristics.

## Introduction

Veterinary healthcare is undergoing rapid changes in both its medical and structural dimensions. In many developed countries, specially in rural areas, veterinary practices encounter difficulties to recruit veterinarians. For example, the Canadian Veterinary Medical Association recently reported that numerous veterinary hospitals are actively looking for an associate veterinarian (1). In a 2019 report in France, the companion animals sector absorbed the vast majority of newly graduated veterinarians to support increased demands for companion animal care (2).

There is good evidence that recruitment efforts need to be made (3) and that the veterinary profession must adapt to new market demands (4–6). Furthermore, feminization is a rapid and increasing change (7). Women represent approximately 55.6% of all veterinary practitioners in France and 76.5% of practitioners that are < 30 years old (8). This phenomenon is apparent in several developed countries. In the US, between 1960 and 2009, the percentage of men in universities decreased from 89 to 22.4% (9). In this new veterinary labor market, there is evidence (10–12) that women earned less than men. This gender wage gap narrowed to 10% (in favor of men) after controlling for various observables in the US in the 1990's (13). Neoclassical economic theory states that workers are paid according to their productivity. Therefore, if men and women of equal productivity do not have equal pay, one can assert that wage discrimination exists. However, this wage gap is not necessarily due to discrimination; alternatively, it may be related to factors that are not observed (14).

Understanding veterinary labor market characteristics facilitates improved decision-making concerning public support for the veterinary healthcare sector. Two important features of the veterinary profession in France are: (i) the increasing proportion of practitioners being employed by a corporate or a large practice (8); and (ii) the rapid shift toward feminization of the profession over the last two decades (15). Additionally, veterinarians can switch from one activity to another with the same diploma (from companion animals to production animals, or between private practice and corporate or public sectors), which generates a risk of supply leakage toward a more attractive activity. Therefore, to match demand and supply, several policies are implemented, notably by governments. In this study, we estimated the gender wage gap in the French veterinary labor market and determined the extent to which this gap can be explained by observable factors.

## Materials and methods

### Data

An anonymous online survey was conducted in November 2021 by the veterinary practitioner union (Syndicat National des Vétérinaires d'Exercice Libéral; SNVEL) in the context of a social management project. In this survey, veterinarians from all sectors (companion animals, food animal and mixed practice) were asked to complete a questionnaire regarding individual characteristics, their work, years of experience, years worked at current job, wage components, and also their feelings regarding their work, their wage, and difficulties encountered (Supplementary Table S1). Associate veterinarians i.e., those holding shares in veterinary practices, were excluded from the survey. Thus the survey included only employees.

### Ordinary least squares method and Mincer's equation

To estimate veterinary wage, we used the OLS method. We relied on Mincer's human capital earnings function, a model that explains the individual wage log as a function of gender, motherhood, level of education, age, years of work experience, and other labor market characteristics. Mincer's (16) model consists of a reduced form model that reflects the equilibrium of supply and demand for labor market characteristics.

In this study, hourly wage was regressed on a set of exogenous variables including gender, on-call services during the week and on the week-end, and veterinarian's tier. The wage considered in the regression was the net hourly salary, excluding premiums. Wage was standardized by number of hours worked, to enable comparisons among declared wages. As the level of education in the veterinary labor market is very homogenous, our model did not account for this variable. We used years of experience as a proxy for veterinarian age. On-calls (week and week-end) correspond to night shifts and week-end shifts and represent additional working hours dedicated to emergencies. For employed veterinarians, five tiers, based on years of professional experience, are used to determine daily salary rates. Tier values ranged from 1 for a veterinarian without professional experience to 5 if the veterinarian held a title of specialist. The tiers 2, 3 and 4 correspond to a veterinarian with <2, between 2 and 4, and > 4 years of professional experience, respectively.

### Estimating gender wage gap by linear regression

To understand the veterinary labor market characteristics and to estimate the gender wage gap, a linear regression was used, as follows:


(1) $$\begin{array}{l} \ln(wage_{i})=\;\beta_{0}+\;\beta_{1}tier_{i3}+\beta_{2}tier_{i4}+\beta_{3}oncallwe_{i[1-12]}\\ \;+\beta_{4}oncallwe_{i[13,+\infty ]}+\beta_{5}oncallw_{i[1-2]}\\ \begin{array}{l} \;+\beta_{6}oncallw_{i[3+\infty ]}+\;\beta_{7}male_{i}+\;\varepsilon_{i} \end{array} \end{array}$$

Where:

ln(*wage*_*i*_) is the logarithm of the net hourly wage of veterinarian i,

*tier*_3_ and *tier*_4_ are dummy variables that represent whether a veterinarian has the 3^rd^ or the 4^th^ tier. The veterinarian's tiers are categorized into three categories (*Tier 2, Tier 3, Tier 4*, with *Tier 2* being the reference category),

*oncallwe*_[1−12]_ and *oncallwe*_[13,+∞]_ are dummy variable for the on-calls in the week-end categorized into three levels (*0, 1 to 12 per year*, and > *13 per year*, with *0* being the reference category),

*oncallw*_[1−2]_ and *oncallw*_[3,+∞]_ are dummy variable for the on-calls during the week categorized into three levels (*0, 1 or 2 per week* and >*3 per week*, with *0* being the reference category),

*male* is a dummy variable taking a value of 1 for men and 0 for women,

and ε_*i*_ is a random error term.

Exogenous variables were selected from among an extended list of variables, with only relevant and significant variables retained. For example, the variable years of experience was excluded because it was highly correlated to the veterinarian's tier. Additionally, other variables (e.g., university where the veterinarian trained) were not significantly correlated to the wage and thus excluded. Our main interest was the parameter β associated to each variable that we wanted to estimate. In this model, estimation of this parameter yielded the effect (by percentage) of an explanatory variable on the wage.

### Estimating wage gap by Blinder-Oaxaca decomposition

The Blinder-Oaxaca (17, 18) decomposition for linear regression model is a technique historically used to estimate gender wage gap. This method divides the wage gap into two groups, an “explained” variation that represents differences in individuals' characteristics and an “unexplained” variation that reflects discrimination or unobserved characteristics.

Denoting male and female by *M* and *F*, the Blinder-Oaxaca decomposition considers the two following equations:


(2) $$\begin{array}{l} wage_{F}=\;\alpha_{F}+\;\beta_{F}X_{F}+\;\varepsilon_{F} \end{array}$$

(3) $$\begin{array}{l} wage_{M}=\;\alpha_{M}+\;\beta_{M}X_{M}+\;\varepsilon_{M} \end{array}$$

Where α denotes the intercept, β the parameters associated to the independent variables, X the independent variables, and ε the normally distributed error term.

To analyze the difference *wage*_*F*_ − *wage*_*M*_, a counterfactual equation is constructed by replacing women's intercept and coefficients by men's intercept and coefficients to obtain:


(4) $$\begin{array}{l} wage_{F}^{*}=\;\alpha_{M}+\;\beta_{M}X_{F}+\;\varepsilon_{F} \end{array}$$

Thus, according to Blinder- Oaxaca decomposition:


(5) $$\begin{array}{l} \bar{wage_{M}}-\bar{wage_{F}}=\bar{wage_{M}}-\;\bar{wage_{F}^{*}}+\;\bar{wage_{F}^{*}}-\;\bar{wage_{F}} \end{array}$$

Where $\bar{wage_{M}}-\;\bar{wage_{F}^{*}}=\;\beta_{M}(\bar{X_{M}}-\bar{X_{F}})$ is the explained difference due to the characteristics and $\bar{wage_{F}^{*}}-\;\bar{wage_{F}}=\left(\alpha_{M}-\;\alpha_{F}\right)+\left(\beta_{M}-\;\beta_{F}\right)\bar{X_{F}}$ is the unexplained difference that may be due to discrimination or to other unobserved characteristics.

## Results

### Descriptive statistics

The sample included 250 observations of veterinarians employed in veterinary clinics. Overall, 61.6% had the fourth tier, there were no Tier 1 veterinarians, and the two Tier 5 veterinarians were excluded (Table 1). Many veterinarians had limited experience (54.3% had <8 years of experience) and many did not have on-calls during the week (48.6%) or during the week-end (40.3%). Overall, 84.7% of veterinarians in our survey were women. Monthly wages ranged from 1,058.24–4,571 euros, with a mean of 2,771.04 euros.

**Table 1:** Descriptive statistics of the sample population.

**Variables**		**Statistics**
Tier (%)	2	13.60
	3	24.80
	4	61.60
Years of experience (%)	<4	34.90
	4–8	29.41
	8–12	20.00
	>12	15.69
On-calls during week-end (%)	0	40.39
	1–12/year	36.86
	>13/year	22.75
On-calls during the week (%)	0	48.63
	1–2/week	46.67
	3–4/week	4.70
Gender (%)	Women	84.71
	Men	15.29
Wage (euros)	mean	2771
	std	619
	min	1058
	25%	2400
	50%	2755
	75%	3191
	max	4571

**Figure 1:**
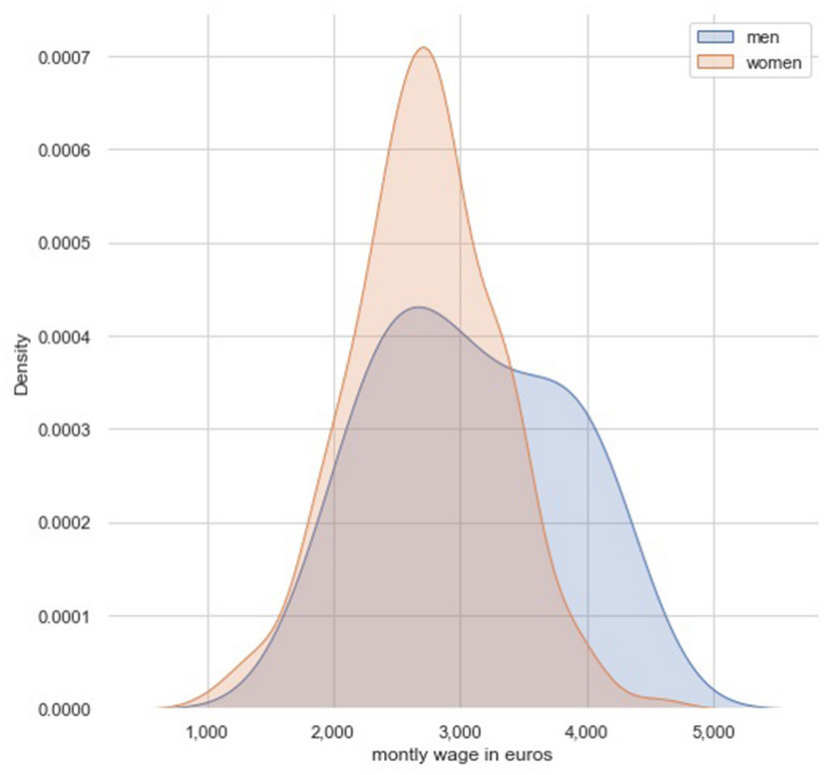
Wage distribution according to gender.

**Figure 2:**
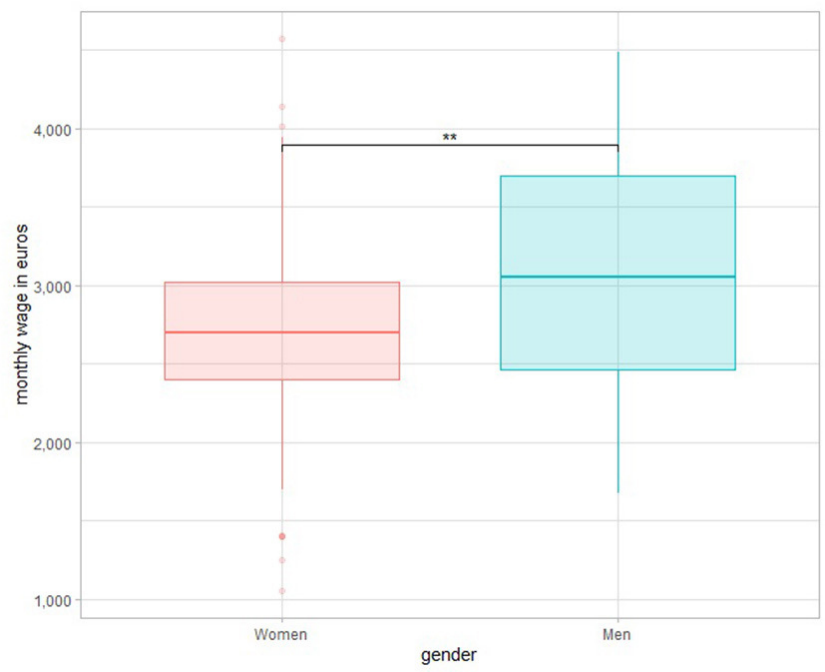
Wage boxplots according to gender. **P < 0.05.

Distribution of wages differed according to gender (Figure 1), men receive more likely a wage > 3,500 euros comparing to women. Based on a Student's *t*-test (Figure 2), the mean of men's wage was greater (*p* < 0.001) than the mean of women's wage.

**Figure 3:**
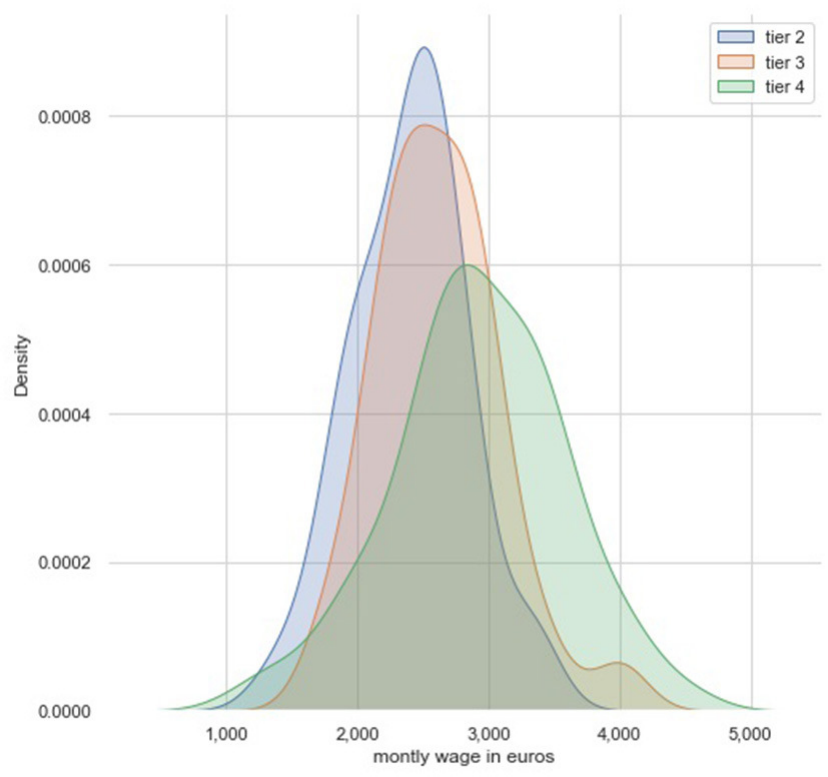
Wage distribution according to tiers.

Wage distributions also differed according to tier, with veterinarians in Tier 4 more likely to be paid >3,000 euros (Figure 3). We performed pairwise paired Student's *t*-tests between the levels of the variable *tiers*. *P*-values were adjusted using Bonferroni multiple testing correction method.

**Figure 4:**
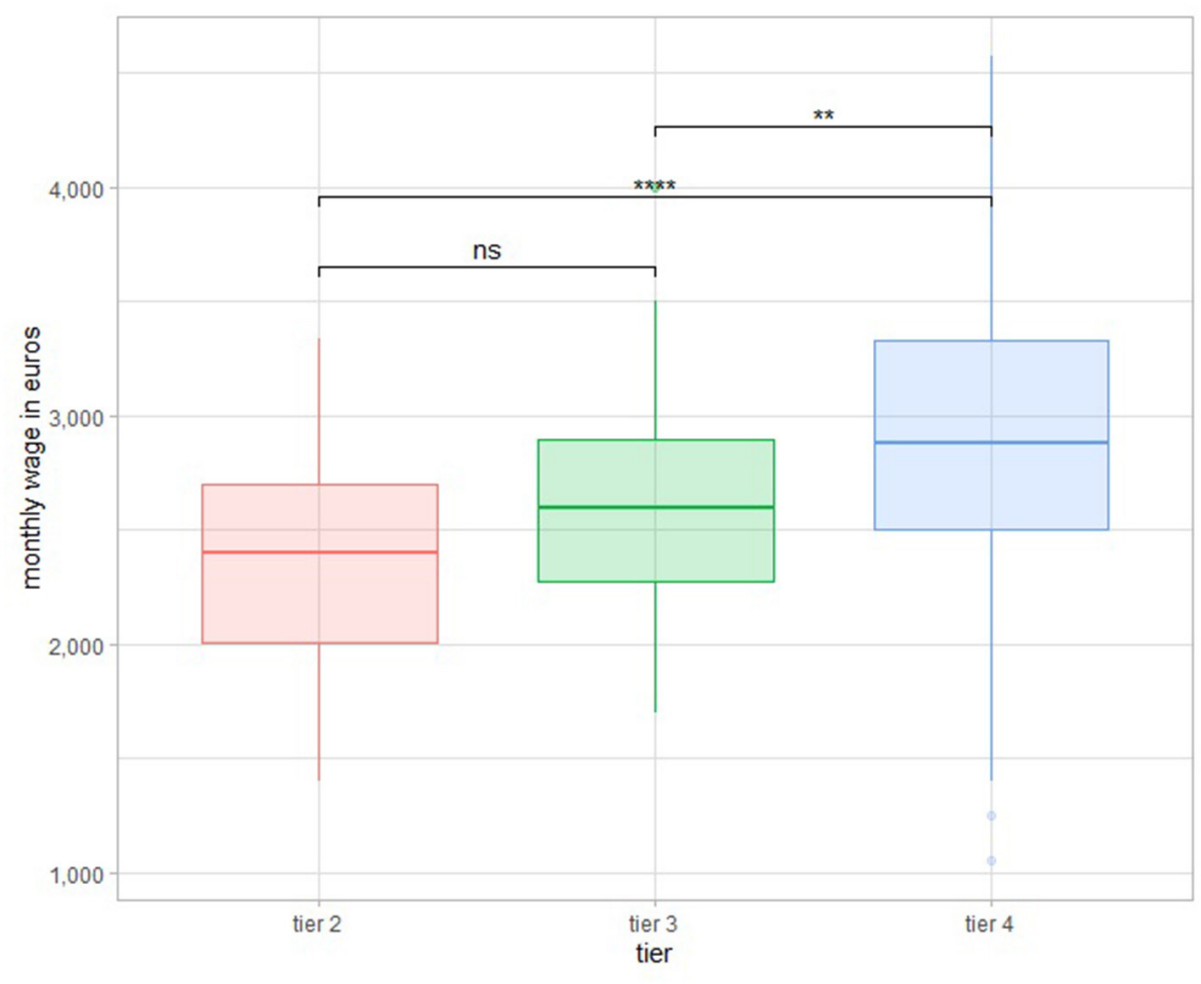
Wage boxplots according to tiers. **P < 0.01; ****P < 0.0001.

There was a difference (*p* < 0.001) between Tiers 2 and 4 and between Tiers 3 and 4 (*p* = 0.00123 < 0.01), except between Tiers 2 and 3 (Figure 4).

Additionally, men were more likely than women to have 0–4 years of experience (46 vs. 33, Supplementary Table S2), whereas more women had 8-12 years of experience (21.8 vs. 10.3%). Furthermore, men were more represented in Tier 2 (26 vs. 11% for women, Supplementary Table S3), whereas women were more represented in Tier 4 (63 vs. 53% for men). Men did more on-calls on week-ends (71.8 vs. 57.4% for women) and during the week (66.66 vs. 49.61% for women, Supplementary Table S3). Veterinarians with <4 years of experience and those with 8–12 years did most on-calls (Supplementary Tables S4, S5).

### Wage characteristics and gender wage gap

Results of OLS regression (1) are in Table 2. The adjusted R-squared of this model was 0.37 (37% of the variability of the hourly wage was explained by the regression model).

**Table 2:** OLS estimation of log wage (in euros) regression.

**Variables**	**Coefficient**	**Standard error**	***t*-test value**	***p*-value**
Intercept	7.6640	0.036	214.395	0.000
tier [3]	0.1159	0.038	3.016	0.003
tier [4]	0.3175	0.034	9.223	0.000
Oncallwe [1–12 per year]	0.1130	0.036	3.176	0.002
Oncallwe [more than 13 per year]	0.0703	0.040	1.774	0.077
Oncallw [1 or 2 per week]	0.0846	0.034	2.484	0.014
Oncallw [3 or 4 per week]	0.0656	0.058	1.127	0.261
Male	0.0929	0.032	2.900	0.004
R-squared	0.39	-	-	-
R-squared adjusted	0.37	-	-	-
Fisher-statistic	17.16	-	-	<0.0001
Goldfeld-Quandt statistic	0.69	-	-	0.98

Source: Author's calculations.

248 observations were included in the regression. The dependent variable is the logarithm of the net hourly wage (in euros). tier [3] and tier [4] are dummy variables for the tier of the veterinarians categorized into 3 categories (tier 2, tier 3, tier 4, with tier 2 being the reference category). *oncallwe*_[1−12]_ and *oncallwe*_[13,+∞]_ are dummy variable for the on-calls in the week-end categorized into 3 levels (0, 1, to 12 per year, and >13 per year, with 0 being the reference category). *oncallw*_[1−2]_ and *oncallw*_[3,+∞]_ are dummy variable for the on-calls during the week categorized into 3 levels (0, 1 or 2 per week and more than 3 per week, with 0 being the reference category). Male is a dummy variable taking a value of 1 for men and 0 for women.

The hourly wage increased for veterinarians with Tier 3 by ~11.6% (*p* < 0.01) compared to veterinarians with Tier 2, controlling for all other variables. Moreover, the wage was ~31.7% higher (*p* < 0.001) for veterinarians with Tier 4 compared to veterinarians with Tier 2 *ceteris paribus*.

Having 1 to 12 on-calls per week-end per year increased wage by 11.3% (*p* < 0.01) *ceteris paribus* compared to veterinarians with no on-call during the week-end. This estimation became 7% (*p* < 0.1) for veterinarians with 13 or more on-calls in the week-end per year. In addition, having 1 or 2 on-calls per week increased wage by 8.5% (*p* = <0.05) *ceteris paribus* compared to veterinarians with no on-call on the week. For veterinarians with three or more on-calls per week, the estimated coefficient was not significant.

Additionally, male veterinarians had wages ~9.3% (*p* < 0.01) higher *ceteris paribus*.

The model was significant (*p* < 0.001). Moreover, the p-value that corresponds to the Goldfeld-Quandt test (*p* = 0.98 > 0.05) leads to failing to reject the null hypothesis of homoscedasticity (i.e., insufficient evidence to say that there was heteroscedasticity).

### Blinder-Oaxaca decomposition

Regarding linear regression, the mens' group was assumed to be the reference coefficient. The estimation of the gender wage gap in Table 3 was decomposed into the explained part relating to differences in veterinarians' characteristics and the unexplained part representing differences for unobservable characteristics or discrimination. The explained variance was not significant (*p* > 0.05) which suggests that when all the characteristics are the same for men and women, the gap was not reduced. Therefore, the gap between men's and women's wage cannot be attributed to observed characteristics. The unexplained part was estimated at approximately 9.3% (*p* < 0.01); therefore, the difference was explained either by a discrimination in the veterinary labor market against women, or by unobservable characteristics in men that are valued by employers or by women's choice.

**Table 3:** Blinder-Oaxaca decomposition of the gender wage gap.

**Variables**	**Estimator** **(standard error)**
Female (average log hourly wage)	4.43
	(0.2195)
Male (average log hourly wage)	4.51
	(0.2419)
Difference	0.0825
	(0.042)
Explained part	−0.0103
	(0.0285)
Unxplained part	0.0928
	(0.0281)

## Discussion

This study estimated and determined factors that influenced wages for French veterinarians in private practice in 2021. We investigated the presence of a wage gap, *ceteris paribus*, between men and women, which may have been a result of a gender discrimination. To that end, we used an OLS method relying on Mincer earning function by regressing the log hourly wage onto exogenous variables including years of experience, gender, on-calls and tier. We used a log transformation of the wage because the dependent variable had a right skew in both cases. The log function made the distribution of the variable more symmetric and more normal and helped to interpret effect of each explanatory variable by percentage, which was more relevant than absolute values.

It was not surprising that the hourly wage increased with both years of experience and with tiers, *ceteris paribus*. In addition, switching from veterinarians without on-calls to veterinarians with on-calls increased the wage, as employers rewarded veterinarians with on-calls.

In our initial dataset, men earned on average 12.3% more than women regardless of characteristics. This estimation was attributed to men doing more on-calls than women. In addition, men are on average older (8) than women in the veterinary labor market; consequently, they have more experience, hence a greater wage. However, this gap simply means that males have better labor market characteristics compared to females, but not higher returns for characteristics that would be interpreted as discrimination. Further analysis has to be made to investigate the gender wage gap when all characteristics are the same.

When men and women with similar characteristics were compared, this gap was adjusted by linear regression (1) and indicated that men earned ~9.3% more than women. Furthermore, there was a similar difference based on Blinder-Oaxaca decomposition. The unexplained difference between men and women wage can be interpreted as the level of discrimination. We performed the BOD method in addition to a linear regression model, as one assumption of linear regression is that the explanatory variables have the same impact on the dependent variable (19). In practice, this assumption is not always verified, and in our case the coefficient associated to the gender estimated by OLS did not always reflect the “true” unexplained part of the gender wage gap. BOD is required when the unexplained part must be estimated.

The reasons underlying this gender wage gap, *ceteris paribus*, have been debated (20–22). The gap that persists after considering the differences in the labor market characteristics of men and women is often interpreted as discrimination. Yet, this result should be interpreted with caution because this gap may be a result of certain unobservable characteristics between veterinarians that employers value (23). This unexplained part can be related to veterinarians' characteristics that cannot be adequately measured as the unusual work hours, an entrepreneurial mindset, attitudes toward risk-taking, or other factors (24). Workforce absences and shorter work hours can explain a significant portion of the wage gap among high-skill occupations (25). In addition, this gap is not necessarily related to employer discrimination because this gap exists even when veterinarians are self employed (14). Furthermore, according to the literature, having children in the household and marital status explains a significant part of the gender wage gap (26). For example, mothers in Germany were willing to compromise for work that provided an opportunity to work from home and with flexible hours (27). The competitiveness can also explain this gender wage gap in career paths (28). It must be noticed that the unexplained part of the gender wage gap could be due to observable factors that are not included in the independent variables as the responsibility assigned to men and women, or of course if it could be caused by discrimination.

Sunstein (29) stated that the labor markets will not eliminate discrimination alone; hence, interventions are necessary to reduce discrimination.

Our study had some limitations. First, the database extracted from the survey contained 84% women, larger than the national percentage (~56.5%) (8). However, veterinarians in our survey were young (84.31% had <12 years of experience (Table 1); therefore, 84.31% were <39 years, as the average age of graduation is 27 years (8). In the same cohort of young veterinarians ranging from 20 to 39 at the national level, most veterinarians were also women (76.58%) (8), which means that our sample was representative of veterinarians <40 years old. Other potential variables explaining the wage gap include practice activity (food animal, companion animal or mixed animal) or practice location; these factors were not investigated in the questionnaire, but may have improved the accuracy of our model.

Overall, the results provided relevant information to understand the main variables that influence veterinarian's wage and to estimate the gender wage gap while controlling for all other variables.

## Conclusions

This study determined the main factors that influence the wages of French veterinarians and provides veterinary professionals and policy makers an overview of characteristics of veterinary labor markets. Additionally, there was clear evidence that there is a gender gap when controlling for all other variables.

## Data availability statement

The data analyzed in this study is subject to the following licenses/restrictions: The data that support the findings of this study are available on request from Laurent lacouture, SNVEL (Syndicat National des Vétérinaires d'Exercice Libéral). Requests to access these datasets should be directed to Laurent lacouture, clinique.lacouture@gmail.com.

## Author contributions

MB and GL are the principal investigators of the study, carried out the conceptualization, the methodology, and the original draft. YN and DR analyzed the data and contributed to the conceptualization. All authors jointly drafted and critically revised the paper, read, and approved the final manuscript.

## Funding

The work was funded by the French Ministry of Agriculture and Food.

## Conflict of interest

The authors declare that the research was conducted in the absence of any commercial or financial relationships that could be construed as a potential conflict of interest.

## Publisher's note

All claims expressed in this article are solely those of the authors and do not necessarily represent those of their affiliated organizations, or those of the publisher, the editors and the reviewers. Any product that may be evaluated in this article, or claim that may be made by its manufacturer, is not guaranteed or endorsed by the publisher.
