# Supplementary material for: The gender wage gap in the French veterinary labor market
Source: Front Vet Sci. 2022 Nov 14;9:1001012. doi: 10.3389/fvets.2022.1001012 (PMC9703345; doi:10.3389/fvets.2022.1001012)
Supplement: Supplementary file 1 [file Data_Sheet_1.docx]

**Supplementary Materials**

Supplementary Table S1: Survey questions

| If you are paid hourly, how many hours per month are scheduled in your contract? |
| --- |
| How many on-calls do you have during the week? |
| How many on-calls do you have during the week-end? |
| How many weeks of holiday do you have per year? |
| How long is an on-call? |
| How many hours did on-calls take? |
| Are you paid in accordance with the collective agreement? |
| What is your tier in the collective agreement? |
| What is your gross basic monthly salary? |
| What is your net salary in the previous 12 months before source taxation |
| Have you had a premium? |
| How much is this premium? |
| How many years have you been practicing? |
| Concerning the on-calls, which day suits you better? |
| In your opinion, how much should you be paid for on-calls? |

Supplementary Table S2: Cross tabulation of gender and years of experience

|  | Women (%) | Men (%) | All (%) |
| --- | --- | --- | --- |
| < 4 years | 32.87 | 46.15 | 34.90 |
| 4 to 8 years | 28.70 | 33.33 | 29.41 |
| 8 to 12 years | 21.76 | 10.26 | 20.00 |
| > 12 years | 16.67 | 10.26 | 15.69 |

Supplementary Table S3: Cross tabulation of gender and tier, on-calls in the week and on-calls in the week-end

|  | Women (%) | Men (%) | All (%) |
| --- | --- | --- | --- |
| Tier 2 | 11.32 | 26.32 | 13.6 |
| Tier 3 | 25.47 | 21.05 | 24.8 |
| Tier 4 | 63.21 | 52.63 | 61.6 |
| No on-call on the week-end | 42.59 | 28.21 | 40.39 |
| 1 to 12 on-calls on the week-end per year | 36.11 | 41.03 | 36.86 |
| More than 13 on-calls on the week-end per year | 21.30 | 30.77 | 22.75 |
| No on-call during the week | 51.39 | 33.33 | 48.63 |
| 1 to 2 on-calls during the week | 44.44 | 58.97 | 46.67 |
| 3 to 4 on-calls during the week | 4.17 | 7.69 | 4.71 |

Supplementary Table S4: Cross tabulation of years of experience and on-calls in the week-end

|  | < 4 years (%) | 4 to 8 years (%) | 8 to 12 years (%) | > 12 years (%) | All (%) |
| --- | --- | --- | --- | --- | --- |
| No on-call | 28.09 | 48.00 | 37.25 | 57.5 | 40.39 |
| 1 to 12 on-calls in the week-end per year | 43.82 | 29.33 | 41.18 | 30.0 | 36.86 |
| More than 13 on-calls in the week-end per year | 28.09 | 22.67 | 21.57 | 12.5 | 22.75 |

Table S5: Cross tabulation of years’ experience and on-calls

|  | < 4 years (%) | 4 to 8 years (%) | 8 to 12 years (%) | > 12 years (%) | All (%) |
| --- | --- | --- | --- | --- | --- |
| No on-call | 37.08 | 57.33 | 47.06 | 60.0 | 48.63 |
| 1 to 2 on-calls during the week | 58.43 | 40.00 | 49.02 | 30.0 | 46.67 |
| 3 to 4 on-calls during the week | 4.49 | 2.67 | 3.92 | 10.0 | 4.71 |
